# Headache as an Adverse Reaction to the Use of Medication in the Elderly: A Pharmacovigilance Study

**DOI:** 10.3390/ijerph18052674

**Published:** 2021-03-07

**Authors:** Cristina Monteiro, Beatriz Dias, Maria Vaz-Patto

**Affiliations:** 1 UFBI—Pharmacovigilance Unit of Beira Interior, Faculty of Health Sciences, University of Beira Interior, 6200-506 Covilhã, Portugal; ufarmabi@fcsaude.ubi.pt; 2 Faculty of Health Sciences, University of Beira Interior, 6200-506 Covilhã, Portugal; zirtaeb96@gmail.com; 3 CICS UBI—Health Sciences Research Centre-UBI, Faculty of Health Sciences, University of Beira Interior, 6200-506 Covilhã, Portugal

**Keywords:** adverse drug reaction, elderly, headache, pharmacovigilance

## Abstract

There is a consensus that elderly individuals are quite vulnerable to adverse drug reactions (ADRs), and headaches are one of the most frequent clinical presentations of central nervous system problems in the general population, which can be an ADR. The purpose of our work was to analyze reports of “headache” associated ADRs in the elderly sent to the Portuguese Pharmacovigilance System (PPS), and also which drugs were more frequently associated with this adverse reaction. A retrospective analysis of suspected ADR reports involving patients aged 65 years or older received by the PPS in the last 10 years was conducted. A search of all the terms associated with the High Level Term “headache” was performed. All duplicate reports were excluded from the analysis. A total of 155 ADRs reports were included, in which 15 reported isolated “headache” as suspected ADR, while the remaining 140 ADRs reports reported “headache” together with several other adverse reactions. Most reports of “headache” ADR occurred in women (74.8%; *n* = 116). About half (46.5%; *n* = 72) of the ADR reports were considered serious. Anti-viral medication, anti-depressants, anti-dyslipidemic agents and central nervous system-acting analgesics were the most frequent drugs associated with “headache” ADR reports in this population. In elderly patients, most ADR reports involving headaches occurred in women and a high percentage (46.5%) were considered serious. Thus, it is important that healthcare professionals pay more attention to headaches reported as ADRs in the elderly and drugs suspected to cause them, in order to increase knowledge about this type of reaction and contribute towards safely using drugs in this age group.

## 1. Introduction

The prevalence of headaches decreases with age, and their characteristics in the elderly tend to be different from those in a younger population [1,2,3,4]. Some headaches are more common in the elderly, such as those associated with ischemic and hemorrhagic strokes, subdural hematomas, trauma, neoplasia, giant cell arteritis and medication overuse [2,3,4]. Primary headache disorders may also manifest in an atypical way in elderly individuals for several reasons. Firstly, elderly individuals may have more prominent migraine-associated visual or sensory phenomena when compared with younger individuals [3]. Secondly, certain headache types tend to be geriatric disorders, such as primary cough headache, hypnic headache, typical aura without headache, exploding head syndrome and giant cell arteritis [2,4]. Although most headaches in the elderly are primary disorders (66%) [5] with no prominent features, serious headaches are more common in this age group, comprising up to 15% of new-onset headaches in this population [5,6].

Elderly patients also have an increased number of comorbidities and, consequently, polypharmacy [7]. The definition of “polypharmacy” is controversial, as the precise number of multiple medications used to define it may vary, most frequently involving at least five or more appropriate drugs [8,9,10,11,12]. An increase in the number of drugs being taken is a risk factor for adverse drug reactions (ADR) and is also associated with increasing ADR-related hospitalizations [11,13]. In fact, ADRs in elderly individuals represent a major burden, causing significant morbidity, mortality and increased health care costs [14]. Elderly patients are particularly susceptible to ADRs not only due to multiple medications but also to comorbidities, cognitive and functional impairment and age-related changes in pharmacokinetics and pharmacodynamics [11,15]. Another frequently observed problem in this population has to do with the use of over-the-counter (OTC) medication that can also interfere with other drugs [16].

There are many drugs, used in acute or chronic treatment of various illnesses, which are associated with headache as an adverse reaction [17,18]. Commonly used drugs in elderly patients that can cause headache include antibiotics (trimethoprim-sulfamethoxazole, tetracycline), sedatives, stimulants, amantadine, levodopa, dipyridamole, β-blockers, calcium-channel blockers, antiarrhythmics, nonsteroidal anti-inflammatory drugs (NSAIDs), H_2_-blockers, bronchodilators, chemotherapeutics (e.g., tamoxifen, cyclophosphamide), hormones and erectogenic agents [2].

Many clinical trials do not recruit elderly patients due to their comorbidities [19]. Thus, treatment recommendations for the elderly are usually based on extrapolation of evidence from trials conducted in healthy and younger populations [9]. Since headaches as a manifestation of ADRs have received limited attention from pharmacovigilance, and there is a well-documented under-representation of elderly in clinical trials [19], it is urgent to study this topic.

Our study aimed to analyze all reports of headache-associated ADR in elderly patients, including serious ADRs, sent to the Portuguese Pharmacovigilance System (PPS), and also describe which drugs were more frequently associated with this reaction. According to the definition of Good Pharmacovigilance Practices, Module VI [20], a serious ADR is “any untoward medical occurrence that at any dose results in death, is life-threatening, requires hospitalization or prolongation of existing hospitalization, results in persistent or significant disability or incapacity, or a congenital anomaly/birth defect”. To the best of our knowledge, this type of study was not performed before, and it may be useful in order to avoid or reduce ADRs in elderly patients.

Our main hypotheses were that headache as an ADR in elderly people increased in Portugal in the last 10 years, and also that sex distribution of this population would be equal, due to the nature of these headaches. Finally we hypothesize that some of these headaches were not innocent and would accompany severe reactions

## 2. Materials and Methods

An observational and retrospective analysis of suspected ADR reports sent to the PPS between 1 January 2007, and 31 December 2017, was performed. This analysis involved only ADR reports of patients aged 65 years or older, using the High Level Term (HLT) “headache”. All duplicate reports were excluded from the analysis, and thus our final sample included 155 reports.

The HLT classification belongs to the Medical Dictionary for Regulatory Activities (MedDRA). This is a rich and highly specific standardized medical terminology developed by the International Council for Harmonisation of Technical Requirements for Pharmaceuticals for Human Use (ICH) which is used by regulatory authorities to facilitate international sharing of regulatory information on medical products used by humans. It is used worldwide by regulatory authorities, global pharmaceutical companies, clinical research organizations and health care professionals to code ADRs [21].

Each report corresponds to a single individual, but each report may correspond to more than one ADR and more than one suspected drug.

In Portugal, the reports are collected by the PPS, coordinated by INFARMED—National Authority of Medicines and Health Products, I.P. They are sent by healthcare professionals, patients/consumers or marketing authorization holders by paper, e-mail, telephone or online.

Patient demographics were analyzed using the following age group distribution: 65–74, 75–84, 85 years or older [12], and gender. Reports were stratified according to their seriousness and source (type of reporter: physicians, pharmacists, nurses, other healthcare professionals, consumers or other non-healthcare professionals or marketing authorization holders). We analyzed all reports with a fatal outcome and the relationship between exposure and death by following the criteria adopted by the PPS and the World Health Organization-Uppsala Monitoring Centre (WHO-UMC) system for standardized case causality assessment [22]. According to this method, which considers the clinical-pharmacological aspects of the report history and the quality of the documentation, ADRs were classified as certain, probable, possible, unlikely, conditional or unclassifiable [22]. The causality assessment was attributed by experts belonging to the PPS.

Using the obtained data, it was also possible to evaluate the polypharmacy rate in this population, considering polypharmacy as the use of five or more drugs per day [10]. It was also checked whether or not suspected ADRs in our study were described in the Summary of Product Characteristics (SmPC) of the respective drug. Drugs involved were categorized by therapeutic group in accordance with the WHO Anatomical Therapeutic Chemical (ATC) classification system [23].

Reports that reported other reactions simultaneously with headaches were also evaluated.

Data were analyzed by descriptive statistics, such as absolute frequency, relative frequency and percentages, using the Office^®^ Excel^®^ 365 software (Microsoft Corporation, Redmond, WA, USA).

## 3. Results

**Figure 1:**
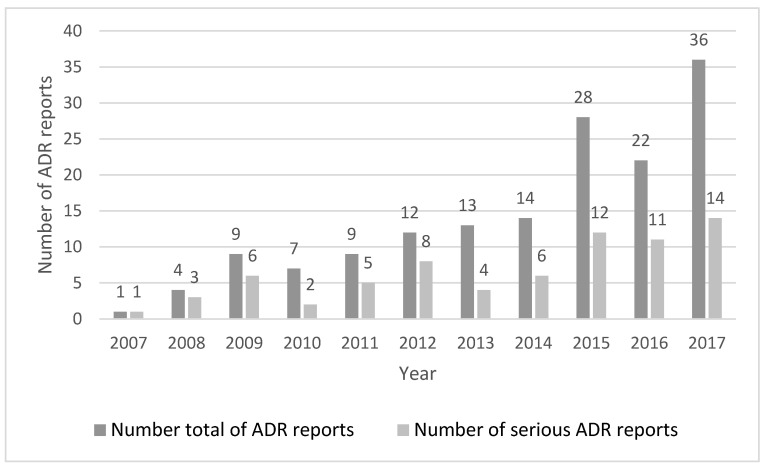
All suspected adverse drug reactions (ADRs) reports versus the serious reports per year.

During the studied 10-year period, we obtained 155 reports in which the HLT term “headache” was an ADR in elderly individuals. In general, there was an increase in reporting over the years, particularly in the last three years, which was also observed in the number of serious ADRs, as can be seen in Figure 1. 

### 3.1. Demographic Data

Of the 155 analyzed reports, most (74.8%; *n* = 116) involved women. The highest number of ADR reports was associated with the group of 65–74 year-old individuals (Table 1).

### 3.2. Prevalence of Polypharmacy 

**Figure 2:**
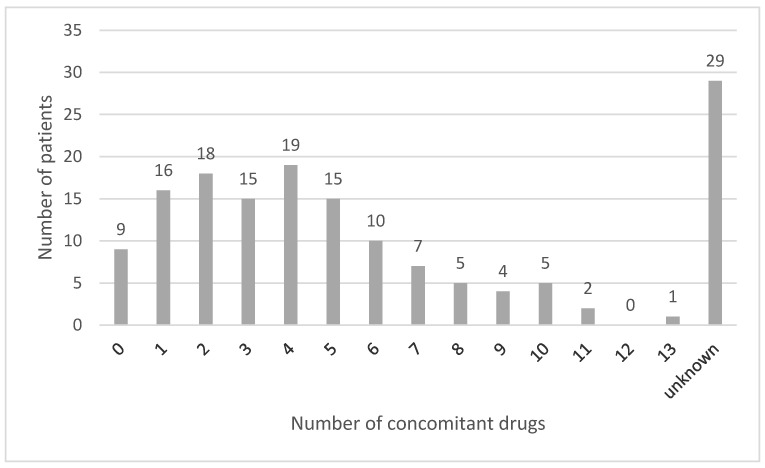
Description of the prevalence of polypharmacy (Note: “unknown” means that it is unknown if the patients were taking concomitant medication or not).

In the majority of the reports (75.5%, *n* = 117) patients were taking one or more concomitant drugs, and in 43.9% of cases (*n* = 68) patients were taking a minimum of five drugs simultaneously. In 29 reports (18.7%), the number of concomitant drugs was unknown, since such information was lacking in the reports. In nine reports (5.8%), patients were not taking any concomitant drugs (Figure 2).

### 3.3. Adverse Drug Reactions Seriousness and Causality

Most of the reports were classified as non-serious (53.5%, *n* = 83). In 72 serious reports (46.5%), 3 had death as an outcome, in 5 reports the patient’s life was threatened, 18 patients were hospitalized or had a prolonged hospitalization and in 27 reports ADRs caused incapacity. Other reports were only considered clinically important.

In the majority of the ADR reports (72.9%), the causal relationship between the drug and “headache” was classified as “possible” or “probable”. In 16.8% of the reports, there was no information about the causality between the drug and ADR. There were only three reports in which ADR was classified as “unlikely” or “unrelated”. These suspected drugs were norfloxacin, nilotinib and *Sabalis serrulatae* fructus.

Moreover, 9.6% of drugs were classified as having a definitive relationship with the ADR by the regulatory authority. In these reports, the involved drugs were alendronic acid and cholecalciferol, aliskiren, fenofibrate, fluoxetine, ginkgo folium, nitroglycerin, normal human immunoglobulin (two cases), propranolol, telmisartan, esomeprazole, tramadol, exemestane and docetaxel.

### 3.4. Drugs Associated with Headache as an Adverse Drug Reaction 

Certain drugs were more frequently suspected as triggers of ADR-headache than others. Table 2 shows the most frequently involved drugs in serious and non-serious ADRs and the number of occurrences for each one.

In 155 reports, we found 177 involved drugs. In 15 reports, more than one drug was suspected as a cause of the ADR-headache. A thorough analysis of drugs involved in serious ADR-headaches was performed. 

### 3.5. Fatal Outcomes

Among the serious ADRs reported, there were three deaths associated with domperidone, enoxaparin and ledipasvir–sofosbuvir. However, after evaluating the reports according to the WHO system for standardized case causality assessment, as described in Methods, it was considered that the suspected drugs were not directly related to the outcome because there were other adverse reactions and complications in the patients’ health status that may have motivated this outcome. As an example, when we searched for details in a report involving enoxaparin, we found that the patient had also suffered a secondary brain hemorrhagic stroke. The cause of death was attributed to stroke, associated with sudden holocranial headache and altered state of consciousness. 

With domperidone, the causality attributed between the drug and the ADRs was only possible. The other ADRs present were hematuria, hematemesis, generalized edema and drowsiness. 

In the report associated with ledipasvir-sofosbuvir, in addition to headache, the patient suffered cardiorespiratory arrest, sepsis, ascites, nausea, vomiting, disorders of the urinary system and abdominal pain. The cause of death was attributed to sepsis and cardiorespiratory arrest, and was thus not drug-related. In conclusion, in these three reports, patients had various previously diagnosed diseases and a poor health status wherefore the expert concluded that the drug was not related to the outcome.

### 3.6. Other Adverse Drug Reactions Concomitant with Headache-ADR

Most described headaches were present simultaneously with multiple other ADRs, such as dizziness, nausea and vision disorders (Table 3). Only in 9.7% (*n* = 15) of the reports was “headache” the single adverse reaction notified.

When associated with vision disorders, reports mostly involved blurred vision, and occasionally double vision, eye pain, ocular photosensitivity, loss of vision, amaurosis fugax and sensation of pressure in the eye. The suspected drugs in these reports were indomethacin and brinzolamide eye drops, two reports with a 35 µg/h transdermal system of buprenorphine, a rectal nitroglycerin ointment and oral glucosamine formulations of 1500 mg, pentoxifylline 400 mg, bisoprolol 2.5 mg, cholecalciferol 22,400 IU, *Escherichia coli* lysate, association of telmisartan and hydrochlorothiazide, acetylsalicylic acid 100 mg, diltiazem 120 mg, nimesulide 100 mg, donepezil 5 mg, levocetirizine 5 mg, aceclofenac 100 mg, celecoxib 100, pregabalin 50 mg and verapamil 120 mg.

In eight reports (5.2%), changes in the state of consciousness were described, namely syncope (four reports), presyncope (three reports), altered state of consciousness (one report) and coma (one report). The drugs involved in the coma were sodium picosulfate combinations and the association of cilazapril and hydrochlorothiazide, both of which are suspected of causing ADRs. For both drugs, the causal relationship was considered probable by the regulatory authority. There was also a case of a fall reported as ADR, which is a relevant event in this older population. This reaction, considered serious, was associated with nitroglycerin and was accompanied by other ADRs such as dizziness and amaurosis fugax. 

Analysis of the SmPCs of the suspected drugs identified in the reports found that headache was not described as a possible ADR in 26 of these drugs. In the remaining drugs (154), this ADR was described with frequencies ranging from very rare to very common. There were also reports in which this adverse reaction was known but its frequency was not known. 

### 3.7. Source of Adverse Drug Reactions Reports

Pharmacists were the health professionals who submitted the highest number of notifications (49.0%, *n* = 76), followed by consumers or other non-healthcare professionals (21.3%, *n* = 33), physicians (15.5%, *n* = 24), marketing authorization holders (11.6%, *n* = 18), nurses (1.9%, *n* = 3) and other healthcare professionals (0.7%, *n* = 1).

## 4. Discussion

Our search involving cases reported to the PPS over a 10-year period retrieved 155 reports of headache as an ADR in elderly individuals under treatment. Most reports of “headache” ADR occurred in women and 46.5% were considered serious. Anti-viral medication, anti-depressants, anti-dyslipidemic agents and central nervous system-acting analgesics were the most frequent drugs associated with “headache” ADR reports in this population.

The population included in the study was mainly composed of female patients in the 65–74-year-old age group, who were polymedicated, which is expected according to data on the elderly population in Portugal that shows that there are more women than men [24]. In addition, studies suggest that women are more susceptible to suffering from headache. A study concerning self-reported headaches across the lifespan in a German sample evaluated headache bouts and analgesic use in old age concluded that elderly women suffer from more frequent episodes of headache than elderly men [25]. This gender issue may also explain findings in our population. Other studies concerning ADRs showed that women are affected twice as much as men due to a combination of pharmacokinetics and pharmacodynamics factors [11], and also because the female gender is associated with an increased risk of an ADR-related hospitalization (RR 1.05; 95% CI 1.03, 1.08) in comparison with males [13]. 

Although the number of reports presented is our study is not too large, it represents the total number of reports sent to the PPS during the last decade in Portugal. In recent years there have been a number of European legislative changes aiming to promote an increase in reporting adverse reactions, whereby the number of notifications of such reactions has been increasing during the observed years. This could also be related to consumer and provider perceptions of how severe an event needs to be to warrant submission of the event report to the post-marketing spontaneous reporting surveillance system, an important tool for monitoring drug safety in a large population [26,27].

Our study shows the importance of headache as an ADR in an older population that is mostly female and polymedicated which may be associated with some serious clinical features. Nair et al. (2016) summarized the available evidence on ADR-related hospital admission in elderly patients living in the community, with a particular focus on risk factors for ADRs leading to hospital admission, and concluded that some of the main risk factors or predictors of ADR-related admissions were advanced age, polypharmacy and comorbidity [11], which may also explain our results. Angamo et al. (2016) evaluated the prevalence and contributing factors to ADR-related hospitalizations in developed and developing countries and concluded that the proportion of severe ADRs in developed countries was twice as high as that in developing countries [15]. The same study concluded that these severe ADRs were associated with an increasingly larger number of older patients with more comorbidities and who were likely taking more medications due to both more advanced age and higher financial capacity to obtain a wider variety of medications [15]. Although the majority of reports in our study were not serious, the percentage of serious cases reported has also been increasing over the years covered by the study, thereby showing the importance of “headache” as a serious adverse reaction particularly in elderly patients [28].

In addition to headaches, several other adverse reactions have been identified. The most common are nausea, vomiting, dizziness and vision disorders. Photophobia, nausea, and vomiting are frequently associated with migraine [29] and occur in approximately one third of patients with the very rare condition of Cardiac Cephalalgia [6]. On the other hand, dizziness and nausea include a spectrum of balance-related adverse reactions with various underlying causes [30]. Even though these ADRs might not represent a direct threat to life, they can indirectly cause secondary damage such as falls and fractures in these elderly patients, and can have serious effects in terms of mobility, cognition and psychological consequences (fear of falling in the future) [31]. Falls can also be a consequence of orthostatic hypotension caused by different drugs in elderly patients [32] and are associated with increased morbidity and mortality in such a population [31,33,34,35]. Nausea and vomiting were also widely reported and, in this population, they carry the risk of dehydration and malnutrition [36]. Although many medications can cause headaches, this ADR can be accompanied by more serious ones, and thus it should not be devalued, particularly in elderly patients. These results are not in agreement with another study performed in this population using a Portuguese pharmacovigilance database, where the most frequently reported suspected ADRs fall within the categories of general disorders and administration site conditions, and skin and subcutaneous tissue complaints [37]. However, our study gives us a global perspective of the ADRs profile in elderly Portuguese patients. In our study, most of the reported ADRs were expected because they are described in SmPC. These results are in agreement with other studies that claim that most ADRs are preventable [38,39]. Organic nitrates were an example of headache-ADR suspected drugs and can produce this ADR because vasodilation is one of the mechanisms that causes headache. This effect is expected to occur when using these drugs; however, it is likely to disappear when treatment is continued because of the development of tolerance [40]. Vasodilatation is often dose-dependent, but may also appear within the therapeutic range. This ADR can occur, namely with cardiovascular drugs such as calcium-channel blockers and angiotensin-converting enzyme inhibitors [17]. Another example involves the use of several antidiabetic drugs which can be related to headaches because of the hypoglycemia that they may induce. This adverse effect results directly from the mechanism of action of the drug and exacerbation of the desired clinical effect (decreased blood glucose) [41]. There are other drug mechanisms that produce headache as an adverse reaction. Raised intracranial pressure is an ADR which is not predictable from the mechanism of action of the medication, such as headache related to drug-induced aseptic meningitis [17]. There are also headaches occurring with chronic medication which are related to raised intracranial pressure as well as headaches related to substance withdrawal [17].

The most frequently notified drugs in this study were antivirals, antidyslipidemic agents, antidepressants, narcotic analgesics, bisphosphonates, drugs used in urine retention, immunomodulators and aromatase inhibitors. Several studies showed that these drugs are responsible for headache as ADR, but some of them are also responsible for a high number of ADRs leading to hospital admission [2,11,17,28,34,37].

Most ADRs were reported directly by healthcare professionals, pharmacists and physicians, as also described in other studies [28]. In our study, pharmacists were the main reporters, just as seen in another study that described the characteristics of voluntarily reported ADRs in a tertiary healthcare setting [41]. Another study of community pharmacists practicing in the West Midlands, UK observed an increasing trend of ADR reporting among community pharmacists [42]. In the same study, the authors concluded that there were three main reasons for under-reporting of ADRs by community pharmacists: consideration that an ADR was not serious enough to report; the perception that well-known ADRs did not need to be reported; and, finally, lack of time to carry out the report [42]. These results emphasize the important role that community pharmacists play in the field of medicinal drugs, namely in terms of drug safety, and also that the abovementioned barriers need to be adequately addressed.

We acknowledge some limitations in our study: ADRs in the PPS were spontaneously reported, and thus the true incidence of ADRs cannot be determined using these data; furthermore, under-reporting is a general problem in pharmacovigilance [26]. On the other hand, ADR reporting may be influenced by several external factors, such as the time that the drug is in the market, litigation and advertising or other media attention and these factors cannot be addressed in our analysis. In addition, it was not possible to classify the different headaches in terms of clinical classification due to the lack of information in the reports.

In spite of the limitations, to the best of our knowledge, our study is the first analysis of “headache” ADR in an elderly population based on data reported to a pharmacovigilance system. The increasing number of elderly individuals with polypharmacy in society and the serious health issues associated with ADR-headache are important factors to evaluate in this population. Our work contributes to shedding some light on this problem, and is also a call to action in order to improve the life of older patients. In addition, we also intend to raise the awareness of the authorities to the need for developing educational programs, either for the population, or for health care providers, with the aim of improving drug safety in elderly people.

## 5. Conclusions

Headache is a serious problem as an adverse reaction to medication in older patients, particularly in women. Many of the events reported in our study were considered serious and were often associated with other serious adverse reactions. For some classes of drugs, a higher association with this kind of adverse effects is clear and such drugs should therefore be prescribed carefully in older populations. The evaluation of ADRs in elderly patients is an important method contributing to prevention of drug-related side effects and promotion of pharmacological safety of this population.

## Figures and Tables

**Table 1:** Characterization of the sample by gender and age.

	Gender			
Age	Female	Male	Unknown	Total
65–74	66	13	2	81
75–84	37	15	-	52
≥85	4	4	-	8
Unknown	9	5	-	14
Total	116	37	2	155

**Table 2:** Anatomical Therapeutic Chemical Classification System code (ATC): drugs most frequently involved in adverse drug reactions (ADRs) and the number of occurrences for each one.

	Serious	Non Serious	
ATC Classification	Drugs (Number of Occurrences)	Drugs (Number of Occurrences)	Total
Antivirals for systemic use	ribavirin (1), sofosbuvir and ledipasvir (2), sofosbuvir (2),	ribavirin (1), sofosbuvir and ledipasvir (4), sofosbuvir (1),	9.0%
Lipid modifying agents	atorvastatin (1), pitavastatin (1)	atorvastatin (1), fenofibrate (1), fluvastatin (1), pravastatin (2), simvastatin (4), simvastatin and ezetimibe (2)	8.4%
Antidepressants	escitalopram(2), mirtazapine (1)	agomelatine (1), fluoxetine (2), mirtazapine (1), sertralina (1), venlafaxine (1), vortioxetine (1)	6.4%
Analgesics	buprenorphine(2), hydromorphone(1), tramadol (1), tramadol and paracetamol (2)	codeine and paracetamol (1), tramadol (1), tramadol and paracetamol (1)	5.8%
Drugs for the treatment of bone diseases	alendronic acid and cholecalciferol (2), zoledronic acid (1)	alendronic acid and colecalcifero (1), alendronic acid (2), ibandronic acid (2)	5.2%
Urologicals	*Sabalis serrulatae* fructus (1), tamsulosin (1)	*Sabalis serrulatae* fructus (2), tamsulosin (1), tamsulosin and dutasteride (1)	3.9%
Immunosuppressants	-	adalimumab (1), etanercept (1), Imatinib (1), mycophenolic acid (1), tocilizumab (2)	3.9%
Endocrine therapy	exemestane (2), letrozole (1)	anastrozole (1), exemestane (1), letrozole (1),	3.9%

**Table 3:** Other adverse drug reactions (ADRs) concomitant with headache-ADR with relative frequency >2%.

Other ADRs Concomitant with Headache	N (%)
Dizziness	29 (18.7%)
Nausea	25 (16.1%)
Vision complaints	24 (15.5%)
Malaise	20 (12.9%)
Vomiting	18 (11.6%)
Myalgia	11 (7.1%)
Diarrhea	11 (7.1%)
Increased blood pressure	11 (7.1%)
Fever	8 (5.2%)
Asthenia	8 (5.2%)
Insomnia	7 (4.5%)
Tachycardia	6 (3.9%)
Fatigue	6 (3.9%)
Stomach ache	6 (3.9%)
Abdominal pain	6 (3.9%)
Arthralgia	5 (3.2%)
Cough	5 (3.2%)
Somnolence	5 (3.2%)
Itching	4 (2.6%)
Ear buzzing	4 (2.6%)
Loss of appetite	4 (2.6%)
Heaviness of head	4 (2.6%)
Dyspnea	4 (2.6%)
Chills	4 (2.6%)
Syncope	4 (2.6%)

## Data Availability

The raw data used in this research are available to the authors, depending on INFARMED’s authorization.
